# Calorie Restriction and Time-Restricted Feeding: Effective Interventions in Overweight or Obese Patients Undergoing Radiotherapy Treatment with Curative Intent for Cancer

**DOI:** 10.3390/nu16040477

**Published:** 2024-02-07

**Authors:** Carmen Vega, Esteban Barnafi, César Sánchez, Francisco Acevedo, Benjamin Walbaum, Alejandra Parada, Nicolás Rivas, Tomás Merino

**Affiliations:** 1 Cancer Center UC, Red de Salud Christus-UC, Santiago 8330032, Chile; cnvega1@uc.cl; 2 Faculty of Medicine, School of Medicine, Pontificia Universidad Católica de Chile, Santiago 8331150, Chile; estebanandres@barnafi.com (E.B.); nicolas.rivass@uc.cl (N.R.); 3 Department of Hematology-Oncology, Faculty of Medicine, Pontificia Universidad Católica de Chile, Santiago 8330077, Chile; csanchez@med.puc.cl (C.S.); fnaceved@uc.cl (F.A.); bvwalbau@uc.cl (B.W.); 4 Department of Health Sciences, Faculty of Medicine, Pontificia Universidad Católica de Chile, Santiago 7820436, Chile; acparada@uc.cl

**Keywords:** intermittent fasting, cancer, curative, radiotherapy, calorie restriction, time-restricted feeding, diet, nutrition, overweight, obesity

## Abstract

This study assesses the feasibility of calorie restriction (CR) and time-restricted feeding (TRF) in overweight and obese cancer patients who realized little to no physical activity undergoing curative radiotherapy, structured as a prospective, interventional, non-randomized open-label clinical trial. Of the 27 participants initially enrolled, 21 patients with breast cancer were selected for analysis. The participants self-selected into two dietary interventions: TRF, comprising a sugar and saturated fat-free diet calibrated to individual energy needs consumed within an 8 h eating window followed by a 16 h fast, or CR, involving a 25% reduction in total caloric intake from energy expenditure distributed across 4 meals and 1 snack with 55% carbohydrates, 15% protein, and 30% fats, excluding sugars and saturated fats. The primary goal was to evaluate the feasibility of these diets in the specific patient group. The results indicate that both interventions are effective and statistically significant for weight loss and reducing one’s waist circumference, with TRF showing a potentially stronger impact and better adherence. Changes in the LDL, HDL, total cholesterol, triglycerides, glucose and insulin were not statistically significant.

## 1. Introduction

In the last decade, cancer and cardiovascular diseases have established themselves as the first cause of morbidity and mortality collectively, with over 18 million new cases of cancer being registered only in 2020 [1]. These pathologies share common risk factors such as a sedentary lifestyle, poor diet, obesity and tobacco and alcohol consumption [2]. Notably, being overweight and obesity are modifiable risk factors present in 74.2% of the Chilean population [3].

Historically, calorie restriction (CR) has been used as a nutritional intervention due to its benefits in weight loss and metabolic health [4]. It is frequently applied in overweight people and, despite showing great results when applied in short-term durations (<6 months), it tends to have many difficulties with adherence when following it for extended periods of time [5].

Alternatives to CR exist, such as the Mediterranean diet or Paleolithic diet [6], and new research has shown more effective interventions for weight loss such as low-carbohydrate diets [7]. Nonetheless, the evidence remains inconclusive due to study limitations such as the duration of trials, sample sizes, or definitions of the different diets [6]. In this context, new nutritional interventions have been explored, such as the ketogenic diet (KD) [8] and intermittent fasting (IF) (defined in Table 1), to measure their impact on weight loss and metabolic health.

IF is one of the most frequently cited diet patterns in 2023 among Americans aged 18–80 years [13]. Despite its popularity, the different approaches to IF (such as TRF, alternate day fasting or a 5:2 pattern) complicate the process of establishing robust scientific evidence.

IF has been shown to be more effective than normal dietary intake for weight loss but equally as effective as CR [14]. A randomized clinical trial (RCT) comparing the KD and IF revealed that while the KD was more effective than IF for short-term weight loss, these patients regained the lost weight in the following 6 months, while those in the IF group had lower weight loss during the intervention but maintained their weights after the followup period [15].

Templeman et al. [16] compared CR, 24 h fasting with 150% energy intake on alternate days and 24 h fasting with 200% energy intake on alternate days. He found alternate day fasting to be less effective than CR for fat mass reduction, with no cardiometabolic improvements.

There are studies which suggest that TRF is better than CR at improving glycemic control [17] and decreasing the time above the range [18], but it also causes a reduction in muscle mass [17], which could be attributed to the amount of protein consumed.

A 2023 review of TRF and specific health conditions found that TRF improved cardiometabolic risk factors and markers of liver fibrosis, improved lean muscle mass when paired with resistance training in patients with sarcopenia and improved insulin resistance independent of weight loss in people with diabetes mellitus type 2 [19], which if paired with weight reduction could be even more beneficial by achieving disease remission [20].

In an attempt to clarify the differences between CR and IF, there is an ongoing clinical trial in patients with polycystic ovary syndrome, but the results are still not available [21].

These ongoing efforts to conclude which interventions are best for weight loss are particularly impactful in patients with cancer, due to the implication of being overweight and obesity in the origin and prognosis [22,23,24] of various types of cancer [25]. Breast cancer is of significant interest as it leads new cancer incidence rates among Chilean women [1].

The pathophysiological reasoning behind the effectiveness of these nutritional interventions has long been researched and explained [26,27,28]. There are preclinical studies that establish the impact from IF and CR on glucose regulation, resistance to stress and inflammation suppression [29]. Despite showing promising results in the regression of tumor volume and radiosensitivity of tumors in mice preclinical models [30,31,32,33], clinical trials are a must to determine the effect of these interventions on patients with cancer [34]. Unfortunately, available evidence on this matter is lacking, and even international guidelines (ACS, NCCN, ESPEN or ASCO) do not have a conclusive stance on their application [22,35,36,37].

The recommendations from these guidelines on breast cancer were reviewed, and they are summarized in Table 2.

A 2020 literature review [38] found 9 clinical trials which focused on radiotherapy and the KD. These studies suggested that the KD may have a beneficial effect, but the small numbers of patients in these cohorts, absence of control groups and KD not being the only variable associated with a response to RT in three studies prevented them from drawing definitive conclusions.

A recent paper from June 2023 by Kalam et al. [39] synthesized results from 23 studies dating from 2020 onward where various approaches to IF were used. Studies involving IF found that the rates of severe toxicity decreased. Those combining fasting and the KD found it was well tolerated and decreased toxicity. Others investigating fasting-mimicking diets (FMDs), a low-calorie diet that is low in protein and carbohydrates but high in unsaturated fat, found it increased ketone bodies and improved plasma glucose but had variable compliance and conflicts of interest. Lastly, TRF showed the highest degree of adherence and helped decrease fatigue and improve visceral adipose tissue, whole-body fat mass, cardiometabolic outcomes, anxiety and depression.

A systematic review on the impact of IF on breast cancer from January 2023 by Anemoulis et al. [40], which included studies from 2009 to 2021, failed to conclude if there were beneficial effects from IF on quality of life (QoL), response after chemotherapy or an improvement in symptoms. They did, however, find that there could be a beneficial effect on chemotherapy-related adverse effects based on markers of DNA and leukocyte damage but required further validation.

A large RCT with 187 patients from 2022 [41] compared intermittent and continous energy restriction during chemotherapy for early breast cancer cases. This study concluded that an intermittent restriction approach had a bigger weight reduction than a continuous restriction when adjusted for total body water. In this cohort, 49.2% of patients with intermittent restriction and 32.7% of patients with continuous restriction underwent a statistically significant weight reduction (defined in the article as >3% weight loss).

The lack of evidence in this area of research and variety of nutritional interventions make it such that it is often overseen and difficult to gauge their real impact. Important international guidelines, such as those the ACS and NCCN published in 2021 and 2022, have no dedicated space for CR or IF with their various approaches in the entirety of their guidelines, whereas the ESPEN or ASCO group them together with other recommendations that, while they may have in common the modification of food intake, have different objectives and methods of implementation.

In practice, many patients show interest in a dietary modification that could increase their chances of success in treatment. A recent study evaluating CR and radiotherapy in breast cancer patients found that up to 75% would be part of a CR study [42]. However, adherence to dietary interventions is variable and can be explained by several reasons, such as depression, stress, previous weight loss attempts or negative perceptions of diet and exercise [43,44]. On the other hand, regular and frequent attendance of nutritional controls, physical exercise and educational materials and feeling in control over what they eat are factors that favor adherence [45,46].

In the growing field of nutritional interventions and cancer, there is still much to be elucidated. The studies we previously mentioned usually opted for one intervention and measured weights and metabolic outcomes. Thus, in this study, we compare two interventions in a similar cohort.

A prospective, interventional, non-randomized open-label clinical trial was built. It assessed the feasibility and effectiveness of CR and TRF in patients with breast cancer who were overweight or obese and were undergoing curative radiotherapy. CR and TRF were chosen due to their history, ease of application and, as previously exposed, evidence supporting them.

The main objective of this clinical trial was to evaluate the feasibility of either CR or TRF in breast cancer patients who are under curative radiotherapy treatment. The secondary outcomes are its effectiveness, as measured by weight loss at the end of the intervention, and changes in several metabolic (HLD, LDL, total cholesterol, triglycerides, glucose and insulin) and anthropometrical parameters (weight and waist circumference).

In this novelty clinical trial, we seek to establish guidelines for future works in this area which can be easily reproducible and improved upon and thus build the foundations for effective and efficient interventions such as TRF or CR.

To our knowledge, this is the first published work of this kind in developing countries, and it shows consistent results in favor of intervention.

## 2. Materials and Methods

We defined a prospective, interventional, non-randomized open-label clinical trial that assessed the feasibility and effectiveness of CR and TRF in patients with breast cancer and a body mass index (BMI) greater than 25 who were treated with curative radiotherapy. The main clinical characteristics of the analyzed cohort can be found in Table 3, and a summary of them is included in Table 4.

The inclusion criteria were as follows:

- ≥18 years old;
- Breast cancer diagnosed by biopsy;
- Overweight or obese, defined as a BMI ≥ 25 kg/m^2^ or 30 kg/m^2^, respectively;
- Indication of radiotherapy with a curative intent.

The exclusion criteria were as follows:

- A BMI <25 kg/m^2^;
- Patients undergoing nutritional treatment or malnutrition;
- Patients with diabetes mellitus using insulin;
- Diagnosis of vascular ischemia, uncontrolled thyroid disease, mental illness without medical supervision, liver disease or malabsorption syndromes (inflammatory bowel disease or celiac disease);
- Patients with a gastric bypass or gastric sleeve;
- Use of corticosteroids or anti-depressants;
- A moderate or high level of physical activity.

The recruitment process was carried out at the “Centro de Cáncer UC CHRISTUS” in Santiago, Chile, where patients with breast cancer are referred for radiotherapy. For their first consultation, if they met our selection process based on the inclusion and exclusion criteria and thus were suitable candidates, then the details of the study along with its risks and benefits were explained. Patients who accepted being part of the study signed informed consent forms and were distributed between CR and IF according to their preferences.

The following steps were taken with each patient to ensure a thorough procedure.

### 2.1. Step 1: Initial Evaluation

- Nutritional evaluation. Before intervention began, baseline measurements of the weight, BMI, waist circumference, and serum levels of glucose, basal insulin and lipids were performed:
  - Weight: was measured with a calibrated scale on a hard surface, with participants removing shoes and heavy clothing and their arms at their sides.
  - Waist circumference was measured with the participant upright with his or her feet close together, arms to his or her sides and abdomen relaxed. The midpoint between the lower margin of the last palpable rib and the top of the iliac crest was located, and measuring tape was wrapped around the waist at this midpoint, ensuring it was horizontal all the way around and not compressing the skin. The participant was asked to breathe out normally before the measurement was taken.
  - The total energy expenditure was estimated through indirect calorimetry.
- Quality of life (QoL) was assessed through the EORTC-c30 and EORTC QLQ-BR23 questionnaires.
- Physical activity was evaluated through the international physical activity questionnaire (IPAQ) from the World Health Organization (WHO) to ensure the participants did not fit the exclusion criteria.
- Regarding eating habits, a 24 h food survey and consumption trend was used to determine the patients’ eating habits (Supplementary Material, “Spreadsheet S1: 24-h Reminder Survey”).

### 2.2. Step 2: Intervention

**Figure 1:**
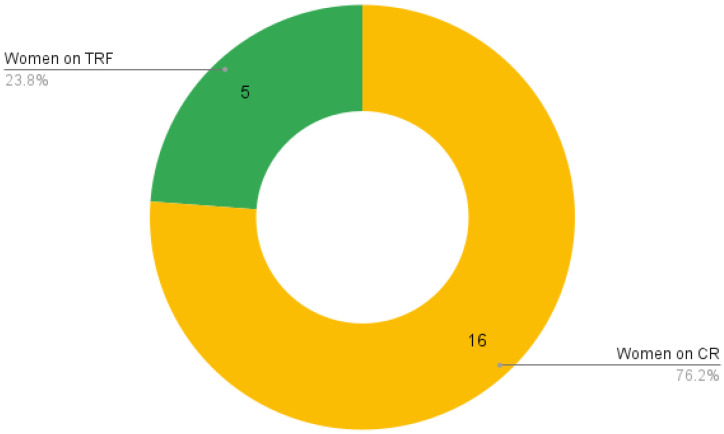
Patient distribution according to their chosen nutritional intervention (TRF or CR).

Each intervention was defined to start 2 weeks before the patient’s oncologic treatment and last for 12 weeks. Once nutritional evaluation was completed, the participants were then assigned to TRF or CR according to their preferences. The patient distribution can be seen in Figure 1.

Nutritional guidelines were made and personalized for each patient according to their group, following certain rules:

- The TRF group followed a diet free of sugar and saturated fats, with caloric intake tailored to the patients’ total energy expenditure. This was distributed over 8 h of food intake and a 16 h daily fast, adjusted to best fit their daily schedules.
- The CR group was based on The Obesity Society guidelines, with a total caloric intake 25% less than the total energy expenditure. This was distributed across four meals and one snack according to the following proportions: 55% carbohydrates, 15% protein and 30% fat, without sugar or saturated fat.

An example of a meal plan can be seen in the Supplementary Materials (“Document S1: Meal Plan”).

### 2.3. Step 3: Monitoring

Throughout the investigation, the following parameters were closely monitored:

- Treatment adherence: Patients were called once a week, and important concepts related to their intervention were reinforced. A dairy registry was considered initially but ultimately decided against due to the anxiety it generated in the patients.
- Toxicity: Once a week during their consultation with radiation oncology, the acute toxicity was assessed using the National Cancer Institute Common Terminology Criteria for Adverse Events (CTCAE).

### 2.4. Step 4: Final Evaluation

Twelve weeks after having started the intervention, and at least 4 weeks after finishing oncologic treatment, the following were measured:

- Acute toxicity;
- Weight and waist circumference;
- Fasting glucose, basal insulin and lipid profile;
- QoL with EORTC-c30 and EORTC QLQ-BR23.

## 3. Hypothesis

CR or TRF are feasible and effective interventions for overweight and obese patients with breast cancer undergoing curative radiotherapy.

## 4. Primary Objectives

We evaluate the feasibility and effectiveness of CR and TRF in overweight and obese patients with cancer under curative radiotherapy.

## 5. Secondary Objectives

- Assess the true adherence, defined as a reduction in body weight of at least 5% compared with the baseline and in at least 50% of the recruited patients;
- Establish which intervention (CR or TRF) has better adherence;
- Assess the impact on quality of life in patients under CR and TRF;
- Assess differences in weight and waist circumference in patients under CR and TRF;
- Assess differences in the serum levels of the fasting glucose, basal insulin and lipid profile for patients under CR and TRF;
- Assess acute toxicity grades ≥2 in patients under nutritional intervention.

## 6. Results

Descriptive statistics with a t test were used to characterize clinical and anthropometric data on the entire cohort. Variables with *p* values < 0.05 were considered statistically significant. Statistical analyses were conducted with STATA/IC (version 16.1, StataCorp, College Station, TX, USA).

**Figure 2:**
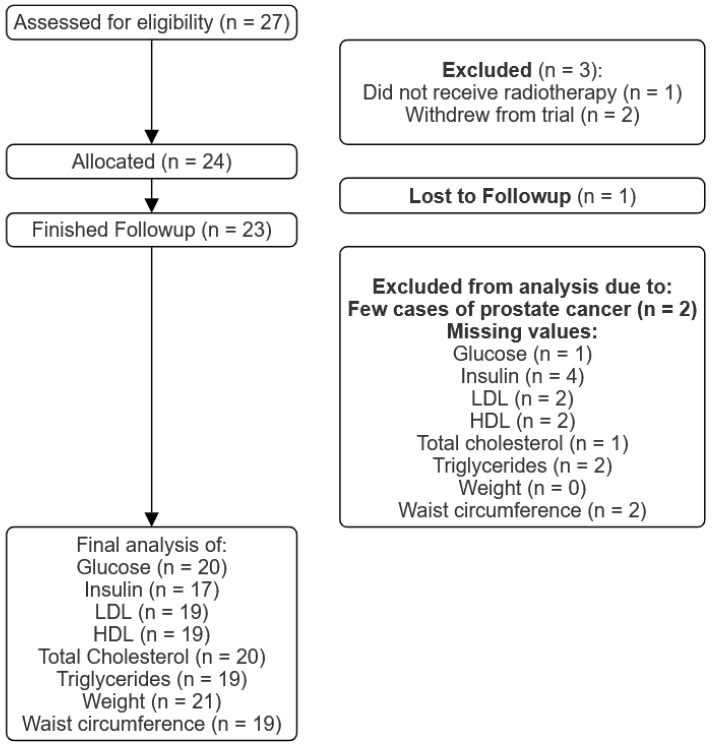
General outline of the study. Diagram depicting steps and results yielded by the study.

Twenty-seven patients were recruited from April 2021 to May 2023. Two patients withdrew from the trial before treatment, and one patient did not receive radiotherapy (as seen in Figure 2). At the end of May 2023, 23 patients had completed 12 weeks of follow-ups, of which 21 were women and 2 were men.

Of the 23 patients available for analysis, 21 were women with breast cancer, with luminal ductal carcinoma being the most common histologic subtype. From the 21 women with breast cancer, 14 received some kind of chemotherapy (neoadjuvant, adjuvant or both), and 19 received endocrine therapy. All 21 patients underwent whole breast radiotherapy (±regional nodal irradiation), and 11 received simultaneous integrated boosts to the tumor bed up to 45.75 Gy.

The remaining two patients were men with prostate cancer. However, they were left out of the analysis due to insufficient scientific significance and difficulty interpreting their results.

The following results were acquired from the data available in Table 5 and Table 6. The findings are described in the following paragraphs.

**Figure 3:**
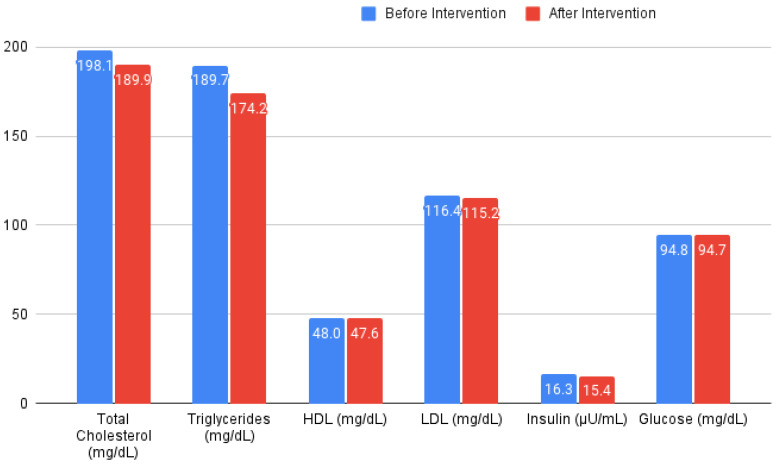
Differences in biochemical measurements before and after intervention. Changes shown in all of the cohorts independent of their chosen interventions.

**Figure 4:**
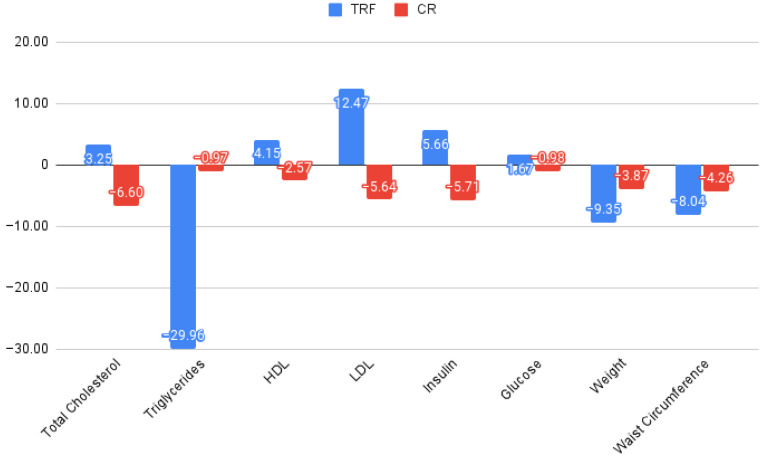
Differences in anthropometric and biochemical measurements between interventions (percentages).

In this study, we evaluated the effects of CR and TRF on various health parameters within each group. An overview can be seen in Figure 3, and the percent change per intervention for each of the measured parameters can be seen seen in Figure 4.

For the total cholesterol, the CR group exhibited a nonsignificant mean increase, while the TRF group showed a nonsignificant mean decrease, indicating considerable variability in the responses within both groups. The triglyceride levels in the CR group slightly increased, whereas the TRF group showed a more substantial increase, yet neither were statistically significant.

The HDL changes were minimal and not significant in either group. The LDL levels also did not show significant changes.

Weight changes were significant in both groups, with the CR group losing an average of 3.02 kg (CI = [0.89, 5.15], *p* value = 0.009) and the TRF group losing an average of 6.84 kg (CI = [2.05, 11.62], *p* value = 0.017), suggesting the effectiveness of both interventions in weight reduction. The waist circumference also significantly decreased (CR: mean change = 4.14, CI = [0.87, 7.41], *p* value = 0.017; TRF: mean change = 7.5, CI = [5.44, 9.55], *p* value = 0.004).

For insulin, the changes were not significant in either group. Similarly, the glucose levels did not exhibit significant changes.

In conclusion, while both the CR and TRF interventions were effective at reducing weights and waist circumferences, their impact on the lipid profile and glucose and insulin levels were not significant. The variability within each parameter suggests a differential response to the dietary interventions.

A percentage analysis of the data from Table 5 and Table 7 was performed, and it yielded the following results:

- There was a reported loss of 5% or more of the initial weight (adherence) in 57.1% of patients (12/21). Thus, both interventions as a whole attained true adherence.
- When evaluating each intervention by itself, only TRF showed true adherence, with 100% of those who chose it adhering to it (5 out of 5), whereas only 43.75% (7 out of 16) of those in the CR group adhered to the intervention.

## 7. Discussion

Our findings show that 57.1% of the patients lost 5% or more of their initial weight, and statistically significant changes were seen in terms of weight and waist circumference. This shows that a nutrition intervention of these characteristics is feasible, attains true adherence and is effective at reducing anthropometric parameters in participants. Changes in the metabolic parameters were not statistically significant.

Our results are consistent with the available literature. TRF showed similar results to the work of Kalam et al. [39], with a high degree of adherence and decrease in weight, and Harvie et al. [41], where IF had a bigger drop in weight compared with CR. However, differences in the mean changes may be explained by the fact that they used another approach of IF which focuses on the amount of calories consumed. The participants in their cohort underwent 2 days of 650–1000 kcal/day before chemotherapy infusion compared with our approach, where we focused on the window of time calories were consumed within.

Of interest are the differences between the findings and the systematic review from Anemoulis et al. [40], where no beneficial effects from IF could be concluded. This could be explained by the type of IF approach utilized in the studies considered in their reviews, where only one of them specified the use of TRF and the others were variations of calorie-restricting approaches of IF. One included Ramadan-fasting patients, and one used an FMD, while the others employed varied hours of fasting before and after chemotherapy. This reinforces the need for clear and established definitions of IF and its different approaches, as well as an urgent necessity for evidence in this field.

Compared with the 2020 literature review from Icard [38], which focused on the KD, our study can draw definite conclusions on the impact on participants’ weights and waist circumferences from TRF and CR.

We found that both TRF and CR managed to impact the weight and waist circumference. We also found that TRF was the superior intervention for weight loss (6.84 kg versus 3.02 kg) and waist circumference reduction (7.5 cm versus 4.14 cm). No statistically significant conclusions could be drawn from changes to the metabolic parameters. The closest parameter to statistical significance was the effect of the use of TRF on triglycerides (with a *p* value of 0.083), but more research is required to further cement these findings in the international literature.

Not only did these interventions as a group achieve true adherence with 57.1% of the participants achieving 5% weight loss, but 100% of the patients within the TRF group showed true adherence, in comparison with only 43.75% adhering in the CR group, despite the previous literature indicating that breast cancer patients favored a CR approach [42]. These findings could be due to how TRF does not restrict the amount of calories in a certain day but rather restricts the timing of meals to 4–12 h a day (8 h in this study), and thus it may be easier to carry on in cancer patients busy with other aspects of the treatment process. Further research is needed on this aspect.

Despite both interventions showing positive results, it seems that TRF as a nutritional intervention has both better adherence and improved anthropometric values. These results show that TRF should be considered as a nutritional intervention in breast cancer patients undergoing curative radiotherapy for the purpose of weight loss.

In this novel clinical trial, we proposed a straightforward and easy-to-replicate method with which to conscript patients and further study nutritional interventions aside from TRF or CR, which we believe can provide the basis for future nutritional intervention studies and open a window into other aspects of nutritional interventions, such as the impact on toxicity reduction which, although seen in animals [47,48], has yet to be reproduced and further explored in human participants.

To our knowledge, this is the first published work of this intervention in developing countries, and it showed consistent efficacy of the intervention.

## 8. Limitations and Future Directions of Work

The limitations of our work are the small sample size and small power for detecting differences in metabolic parameters, a relatively short period of surveillance, a lack of adherence by patients in the CR group to the guidelines given, limited generalization of the study due to only conscripting breast cancer patients, only evaluating the TRF approach of IF, the lack of long term followups, and the absence of a control group.

This work could be improved by an increase in the number of patients, including QoL and acute toxicity to create a more holistic view of the patient outcomes, including other cancer populations to further generalize our findings, increasing the time of surveillance, including a period of followups, employing other nutritional interventions such as the KD, alternate day fasting, or the FMD to better compare the results between interventions, measuring the blood levels of inflammatory response markers, measuring the blood levels of hormonal markers, measuring the fat mass and lean mass percentages, the inclusion of a control group, or the incorporation of feedback from the participants.

We believe these factors must be taken into account to ensure robust findings. Once international consensus is achieved between the different definitions and approaches of the various nutritional interventions, and a strong methodology is properly built and assured, future research should look to apply these conditions to different cohorts to better generalize these findings and eventually include them in international guidelines.

## 9. Conclusions

Cardiovascular diseases and cancer lead incidence and mortality rates worldwide, sharing common risk factors such as being overweight and obesity. These play an important role in the origin of breast cancer, and thus different interventions have been investigated to establish a faithful and reproducible way to achieve weight loss in cancer patients.

The theory behind the benefits of diet and weight loss in cancer has been largely studied and established in preclinical studies, but clinical studies that show these results in practice are few and far between and limited in their approach. This could be attributed to the numerous interventions in existence and a lack of agreement on definitions to establish which ones are best and also a lack of patients through which these interventions have been measured thoroughly.

Two of these interventions are TRF and CR. These nutritional interventions were chosen in this study due to their historical relevance and the evidence supporting them. Both CR and TRF achieved statistically significant weight loss and reductions in waist circumferences, but TRF showed a larger mean change in both weight loss and reductions in waist circumferences. Changes in the metabolic parameters were not statistically significant. Our findings are consistent with the literature. It can be concluded that TRF should be preferred over CR as a nutritional intervention for overweight or obese breast cancer patients undergoing radiotherapy treatment with curative intent.

Differences with the literature can be explained by the heterogeneity in the application of IF and its different approaches, lacking a formal definition. This leads to large differences in the fasting hours, number of fasting days, and the existence or nonexistence of a caloric restriction.

We propose a series of simple-to-apply and reproducible steps through which the metabolic parameters and anthropometry can be measured. We hope this helps establish concrete and well-established definitions of the various fasting regimes and motivates other researchers to further investigate and build the foundation of what we believe is a low-cost and effective way to lose weight and improve metabolic parameters.

## Figures and Tables

**Table 1:** Nutritional intervention definitions and different approaches to IF [5]. In this study, we analyzed CR and IF, specifically the time-restricted feeding (TRF) approach. We also define other approaches so the reader understands the crucial differences between nutritional interventions and the key aspects of the various approaches to IF.

Intervention	Approach	Definition
Intermittent Fasting		Periods of voluntary abstinence from food and drink [5]. There are various ways to approach IF. Most of them have no caloric restriction associated or have caloric restriction for short periods of time.
	TRF	Fasting that requires limiting the consumption of calories to a window of time, typically between 4 and 12 h daily.
	5:2	Two days per week, for 24 h each day, an extremely low-calorie diet is applied.
	B2	Two large meals are eaten per day: breakfast between 6:00 a.m. and 10:00 a.m. and lunch between 12:00 p.m. and 4:00 p.m. No dinner.
Calorie Restriction		Nutritional intervention where the focus is on reducing energy intake but keeping adequate nutrition [9]. In general, 10–30% restriction is accepted as having beneficial effects and being tolerable [10,11].
Ketogenic Diet		Diet primarily consisting of high fat intake, moderate protein consumption and low carbohydrate intake. The macronutrient distribution typically ranges from approximately 55% to 60% fat, from 30% to 35% protein and from 5% to 10% carbohydrates [12].

**Table 2:** Summary of recommendations from international guidelines: the American Cancer Society (ACS)), National Comprehensive Cancer Network (NCCN), European Society for Clinical Nutrition and Metabolism (ESPEN) and American Society of Clinical Oncology (ASCO).

ACS	Published in 2022. Focuses mostly on body mass index (BMI), dietary patterns, specific food avoidance and physical activity.
NCCN	Published in 2022. Similar to the ACS, it focuses mostly on BMI, dietary patterns, specific food avoidance and physical activity. No mention of intermittent fasting or caloric restriction at all in their guidelines.
ESPEN	Published in 2021. Does not recommend fasting unless there is evidence of a benefit.
ASCO	Published in 2022. Mentions intermittent fasting as an intervention but refrains from recommending it as there is insufficient evidence, and points to 5 systematic reviews. These reviews revised 177 articles, where only 7 talked about any kind of weight loss intervention, and of these, 5 of them investigated short-term fasting, finding it to be well tolerated and have beneficial effects.

**Table 3:** Clinical characteristics of patients.

Age	Cancer Type	cTNM	pTNM	Stage	EEE	Intervention
30	IDC—Luminal	cT2N1M0	ypT1N1M0	IB	2096	CR
34	IDC—TN	cT3N1M0	ypT1bN0M0	IIA	1922	CR
67	IDC—Luminal	cT1N0M0	pT2N3M0	IIIB	1977	CR
43	ILC—Luminal	cT1cN1M0	pT2mfN1M0	IB	2418	CR
53	Poorly Diff.—Luminal	cT2N1M0	ypT0?N0M0	ND	2085	CR
48	IDC—Luminal	cT2N1M0	ypT1N0M0	IB	1956	CR
70	IDC—Luminal	cT1N0M0	pT1cN0M0	IA	1818	CR
58	IDC—TN	cT2N2M0	ypT0N0M0	ND	2017	CR
36	IDC—Luminal	cT2N0M0	pT2N0M0	IB	2014	CR
49	IDC—Luminal	RB cT1N1; LB T1N0	RBypT0N0M0; LBypTisN1	IIA	2142	CR
65	ILC—Luminal	cT2N0M0	pT3N0M0	IIIA	1681	CR
43	IDC—Luminal	cT2N0M0	pT2N1M0	IB	2112	CR
60	IDC—Luminal	ND	pT2N1M0	IB	1800	CR
47	IDC—Luminal	cT1N0M0	pT1N0M0	IA	1621	CR
60	IDC—Luminal	cT1N0M0	pT1N1M0	IB	1944	CR
52	IDC—Luminal	cT1N0M0	pT1N1M0	IB	ND	CR
49	DCIS—Luminal	cTisN0M0	pTisN0M0	0	2000	TRF
62	IDC—Luminal	cT1N1M0	ypT2N1M0	IB	1736	TRF
55	IDC—TN	cT4bN0M0	pT0N0M0	ND	1922	TRF
48	IDC—Luminal	cT1N0M0	pT2N1M0	IB	2092	TRF
65	ILC—Luminal	cT1N0M0	pT2N1micM0	IB	1871	TRF

IDC = invasive ductal carcinoma; ILC = invasive lobular carcinoma; DCIS = ductal carcinoma in situ; TN = triple-negative breast cancer; EEE = estimated energy expenditure; ND = no data; RB = right breast; LB = left breast.

**Table 4:** Summary of clinical characteristics of patients.

Number of Patients		*n* = 21
Mean Age (years)		52
Cancer Type	IDC	16
	ILC	3
	DCIS	1
	Poorly Diff.	1
	Luminal	18
	TN	3
Stage	0	1
	1	13
	2	2
	3	2
Intervention	CR	16
	TRF	5

IDC = invasive ductal carcinoma; ILC = invasive lobular carcinoma; DCIS = ductal carcinoma in situ; TN = triple-negative breast cancer; CR = calorie restriction; TRF = time-restricted feeding.

**Table 5:** Table with metabolic parameters and their values before and after intervention for each patient.

		Total Cholesterol (mg/dL)		Triglycerides (mg/dL)		HDL (mg/dL)		LDL (mg/dL)		Insuline (μU/mL)		Glucose (mg/dL)	
Age	Group	Baseline	Final	Baseline	Final	Baseline	Final	Baseline	Final	Baseline	Final	Baseline	Final
30	CR	176	191.0	143	296.0	43	44.0	105.0	87.0	15.3	21.9	91	84.0
34	CR	244	214.0	236	157.0	51	56.0	146.0	126.6	9.9	11.8	87	95.0
67	CR	204	203.0	174	137.0	38	43.0	132.0	132.0	10.0	11.6	106	96.0
43	CR	169	207.0	133	112.0	49	64.0	93.0	121.0	13.0	8.8	85	78.0
53	CR	158	110.0	428	317.0	37	37.0	121.0	99.0	43.2	47.8	108	112.0
48	CR	160	137.0	212	334.0	35	30.0	82.0	41.0	15.4	18.7	102	103.0
70	CR	149	128.0	77	84.0	44	29.0	89.0	83.0	22.1	12.5	103	101.0
58	CR	182	184.0	241	228.0	38	34.0	96.0	104.0	10.0	11.9	106	113.0
36	CR	188	202.0	82	68.0	67	82.0	105.0	106.0	5.4	4.7	87	92.0
49	CR	163	171.0	81	90.0	60	58.0	86.0	95.0	15.3	18.1	96	91.0
65	CR	222	188.0	166	181.0	56	52.0	133.0	99.0	6.5	11.1	88	91.0
43	CR	204	168.0	356	179.0	22	26.0	111.0	107.0	67.8	17.3	102	85.0
60	CR	241	ND	180	ND	60	ND	145.0	ND	11.6	ND	81	ND
47	CR	244	280.0	76	127.0	56	65.0	172.8	189.0	7.8	ND	81	89.8
60	CR	230	188.0	360	404.0	43	33.0	115.0	155.0	13.6	15.4	91	75.0
52	CR	243	211.0	187	ND	67	ND	139.0	ND	9.3	ND	96	96.0
49	TRF	214	167.0	230	138.0	40	46.0	128.0	93.0	14.0	7.6	89	98.0
62	TRF	211	241.0	150	114.0	46	48.0	135.0	170.0	10.8	14.1	99	106.0
55	TRF	175	183.0	104	87.0	54	60.0	100.0	105.0	10.1	9.2	92	89.0
48	TRF	192	253.0	132	135.0	48	40.0	117.6	185.0	15.3	22	106	104.0
65	TRF	192	172.0	235	122.0	53	57.0	92.0	91.0	ND	13.4	94	91.0

CR = calorie restriction; TRF = time-restricted feeding; ND = no data.

**Table 6:** Analysis of the impact of TRF and CR on metabolic and anthropometrical parameters. Std Dev = standard deviation; CI = confidence interval.

Parameter	Group	Mean Change	Std Dev	95% CI	*p* Value
Total Cholesterol	CR	10.27	28.11	[−5.30, 25.83]	0.179
	TRF	−6.4	42.09	[−58.67, 45.87]	0.751
Triglycerides	CR	3.64	85.51	[−45.73, 53.02]	0.876
	TRF	51	49.553	[−10.53, 112.53]	0.083
HDL	CR	1.00	8.64	[−5.99, 3.99]	0.672
	TRF	−2	5.83	[−9.24, 5.24]	0.486
LDL	CR	−3.01	22.77	[−10.13, 16.16]	0.629
	TRF	−14.28	38.73	[−62.37, 33.81]	0.457
Weight	CR	3.02	3.99	[0.89, 5.15]	0.009
	TRF	6.84	3.85	[2.05, 11.62]	0.017
Waist Circumference	CR	4.14	5.67	[0.87, 7.41]	0.017
	TRF	7.5	1.66	[5.44, 9.55]	0.004
Insulin	CR	2.76	14.94	[−6.26, 11.79]	0.518
	TRF	−0.675	5.649	[−9.66, 8.31]	0.826
Glucose	CR	1.81	8.30	[−2.78, 6.41]	0.412
	TRF	−1.6	5.90	[−8.92, 5.72]	0.577

**Table 7:** Anthropometry values before and after intervention. Also included is the percent change in weight, which was used to measure adherence.

		Weight (kg)			Waist Circumference (cm)	
Age	Group	Baseline	Final	% Change	Baseline	Final
30	CR	82.8	78.1	−5.68	91	86.0
34	CR	70.0	63.0	−10.00	84	79.0
67	CR	83.5	89.0	6.59	107	116.0
43	CR	103.0	100.0	−2.91	ND	ND
53	CR	82.5	83.5	1.21	104	101.0
48	CR	72.5	71.6	−1.24	93	93.5
70	CR	72.3	63.5	−12.17	97.5	88.0
58	CR	77.0	75.0	−2.60	108	101.0
36	CR	76.8	72.0	−6.25	85.5	81.0
49	CR	86.2	86.0	−0.23	96.5	95.0
65	CR	62.6	60.5	−3.35	88	85.0
43	CR	84.0	73.3	−12.74	98	90.5
60	CR	71.0	66.0	−7.04	96	ND
47	CR	67.0	68.0	1.49	89	90.0
60	CR	81.2	77.0	−5.17	102	95.0
52	CR	77.0	74.6	−3.12	107.5	92.0
49	TRF	75.8	62.3	−17.81	92	83.0
62	TRF	66.5	61.0	−8.27	102	96.0
55	TRF	70.0	66.0	−5.71	85	79.0
48	TRF	77.4	72.8	−5.94	93	86.0
65	TRF	76.1	69.5	−8.67	94.5	85.0

## Data Availability

All statements and conclusions made within this article have been made with the data and information contained within the provided supplements (tables and figures). No other data were used.

## Supplementary Materials

The following supporting information can be downloaded at https://www.mdpi.com/article/10.3390/nu16040477/s1. The following supporting information has been uploaded together with the manuscript. Spreadsheet S1: 24 h reminder survey. Document S1: Meal plan.

## Abbreviations

The following abbreviations are used in this manuscript:

CR	Calorie restriction
TRF	Time-restricted feeding
KD	Ketogenic diet
IF	Intermittent fasting
RCT	Randomized clinical trial
ACS	American Cancer Society
NCCN	National Comprehensive Cancer Network
ESPEN	The European Society for Clinical Nutrition and Metabolism
ASCO	American Society of Clinical Oncology
BMI	Body Mass Index
FMD	Fast-mimicking Diet
QoL	Quality of Life
IDC	Invasive ductal carcinoma
ILC	Invasive lobular carcinoma
DCIS	Ductal carcinoma in situ
TN	Triple-negative breast cancer
EEE	Estimated energy expenditure
ND	No data
RB	Right breast
LB	Left breast
IPAC	International Physical Activity Questionnaire
WHO	World Health Organization
CTCAE	Common Terminology Criteria for Adverse Events
Std Dev	Standard deviation
CI	Confidence interval
