# Supplementary material for: Calorie Restriction and Time-Restricted Feeding: Effective Interventions in Overweight or Obese Patients Undergoing Radiotherapy Treatment with Curative Intent for Cancer
Source: Nutrients. 2024 Feb 7;16(4):477. doi: 10.3390/nu16040477 (PMC10892811; doi:10.3390/nu16040477)
Supplement: Supplementary file 1 [file nutrients-16-00477-s001.zip › Document S1. Meal Plan CR.pdf]

Meal Plans (Caloric restriction group)

Breakfast 9:00-10:00am
1 to 2 cups of coffee (+-a finger of skim milk + water) with sucralose or stevia
Wholemeal bread 1 unit or marraqueta bread 1/2 unit without crumbs or 1 small wholemeal pita bread or ciabatta bread half a unit
Add to bread: 1 egg (with a little oil), a tablespoon of avocado + free tomato, turkey or chicken ham.

Snack 12.00
Tea with sucralose or stevia, without sugar or coffee with sucralose or stevia + light yogurt or
a fruit, preferably apple, orange, pear, strawberry, raspberry, blueberry (1 cup)

Lunch 1 p.m.
Meats: consume a piece the size of your hand or a plate of tea
Red met= 1 to 2 times per week
White meat (skinless chicken) = 3 times a week
Fish: salmon, horse mackerel, tuna.
Meats must be without sauces or creams, nor should they be breaded or fried, steamed or grilled, in juice or roasted.
Vegetables: Corn, beans, peas, carrots, beets, potatoes = one tablespoon, daily
Chicory, celery, onion, asparagus, spinach, lettuce, cucumber, radish, cabbage, chard, artichoke, eggplant, broccoli, cauliflower, green beans, tomato, hearts of palm, mushrooms, pickled cucumbers: free consumption.
Cereals: legumes, rice, noodles, preparations based on potatoes or pasta, carbonada, charquicán: half a cup cooked, 2 times a week
Dessert: light jelly
Liquids: Water or light juice (powder): a large glass

Snack 3:00 p.m.
Light yogurt or
A fruit, preferably apple, orange, pear, strawberry, raspberry, 1 cup of blueberry 1 cup

Dinner 7:00 p.m.
Meats: consume a piece the size of your hand or a plate of tea, cooked
White meat (skinless chicken) = 3 times a week
Fish: salmon, horse mackerel, tuna.
Vegetables: Corn, beans, peas, carrots, beets, potatoes = one tablespoon, daily
Chicory, celery, onion, cochayuyo, asparagus, spinach, lettuce, cucumber, radish, cabbage, chard, artichoke, eggplant, broccoli, cauliflower, green beans, tomato, hearts of palm, mushrooms, pickled cucumbers: free consumption
Cereals: legumes, rice, noodles, preparations based on potatoes or pasta, carbonada, charquicán: half a cup cooked, 2 times a week
Dessert: Light jelly

Snack 9: p.m
Light jelly: free consumption
Light yogurt
