# Supplementary material for: Calorie Restriction and Time-Restricted Feeding: Effective Interventions in Overweight or Obese Patients Undergoing Radiotherapy Treatment with Curative Intent for Cancer
Source: Nutrients. 2024 Feb 7;16(4):477. doi: 10.3390/nu16040477 (PMC10892811; doi:10.3390/nu16040477)
Supplement: Supplementary file 1 [file nutrients-16-00477-s001.zip › Spreadsheet S1. 24 hour survey.pdf]

<u>24-hour Reminder Survey</u>

Name of patient: ____
Age____________ Sex: ________________ Weight:________ Height:________ BMI:_________
Physical Activity: _______________________ Frequency of Physical Activity: _______________________

24 HOUR REMINDER SURVEY						
**TIME**	**FOOD MINUTE**	**INGREDIENTS**	**MEASURES**	**QUANTITY (IN GRAMS)**	**OBSERVATIONS**	**PORTION EXCHANGE LIST**
